# P-1770. Epidemiological Trends of Kudoa septempunctata Food Poisoning in Japan, 2013–2023

**DOI:** 10.1093/ofid/ofaf695.1940

**Published:** 2026-01-11

**Authors:** Yoshiro Hadano, Hirotake Mori, Yuichiro Tanaka, Aongart Mahittikorn, Satoshi Ohno

**Affiliations:** Shimane University Hospital, Izumo, Shimane, Japan; Juntendo University Faculty of Medicine, Bunkyo-ku, Tokyo, Japan; Shimane University, Izumo, Shimane, Japan; Department of Protozoology, Faculty of Tropical Medicine, Mahidol University, Bangkok, Krung Thep, Thailand; Shimane University Hospital, Izumo, Shimane, Japan

## Abstract

**Background:**

*Kudoa septempunctata*, a parasite found in olive flounder, poses a growing food safety risk in East Asia, particularly Japan and South Korea. Linked to raw fish consumption, *K. septempunctata* poisoning causes brief gastrointestinal symptoms. This study aimed to characterize recent epidemiological trends and characteristics of *K. septempunctata* food poisoning by using national data from Japan.

**Methods:**

This retrospective study examined *Kudoa* food poisoning cases reported in Japan between January 2013 and December 2023. Data from the Ministry of Health’s “Foodborne Illness Statistical Data” report were assessed for case counts, outbreaks, and implicated foods.

**Results:**

A total of 2,009 cases were reported, peaking in 2014 (429 cases) and declining to < 100 cases since 2020. October had the highest number of monthly reports. The age distribution showed that the majority of cases occurred among older adults, with individuals aged 60–69 years (23.5%) and those 70 years and older (26.0%) together accounting for nearly half of all cases. Cases among individuals younger than 20 years comprised less than 2.5% of all cases. Flounder, particularly sashimi and sushi, were implicated in 99% of cases. The highest case counts occurred in Yamaguchi, Osaka, and Fukuoka prefectures (160, 155, and 154, respectively). Tottori, Shimane, Yamaguchi and Oita prefectures had the highest incidence rates (14.3, 10.9, 10.7, and 10.7 per 1,000,000 population, respectively). Prefectures along the Sea of Japan tended to report higher incidence rates.

**Conclusion:**

his is the first study to describe *Kudoa* food poisoning in Japan. Clinicians should consider *Kudoa* infections in cases of food poisoning involving raw fish.

**Disclosures:**

All Authors: No reported disclosures

